# Smartphone use by government dermatology practitioners in Kuwait: a self-reported questionnaire based cross-sectional study

**DOI:** 10.1186/s12911-019-0883-z

**Published:** 2019-08-05

**Authors:** Ali Jasem Buabbas, Prem Sharma, Adel Al-Abdulrazaq, Hashem Shehab

**Affiliations:** 1 Community Medicine, Faculty of Medicine, Kuwait University, P. O. Box 24923, 13110 Safat, Kuwait; 2 Biostatistics, Dasman Diabetes Institute, P. O. Box 1180, 25462 Dasman, Kuwait; 3 Dermatology Consultant, Al-Adan Hospital, Al-Ahmadi Health Region, Kuwait; 4 Dermatology Trainee, Asad Alhamad Dermatology Center, Al-Sabah Health Region, Kuwait

**Keywords:** Smartphone technology, Dermatology practice, Clinical photography, Dermatologist perspectives

## Abstract

**Background:**

The potential for smartphones to revolutionize the way that medical doctors practice has become a reality, particularly in specialities where visual examination is a principal step in assessing a medical case, like dermatology. Smartphones as devices hold similar capabilities to personal computers and laptops and could play an important role in supporting medical practitioners in clinical practice at the point of care and beyond. This study aimed to assess the role of smartphone technology use in dermatology practice in Kuwait, together with the potential of digital photography and users’ concerns.

**Methods:**

This cross-sectional survey involved a population of dermatology practitioners of all levels working in 11 dermatology centers distributed across six health regions in Kuwait. A validated self-reported questionnaire was used to collect data from the participants about their smartphone use. Quantitative analytical methods were undertaken to analyse the questionnaire responses.

**Results:**

A total of 210 dermatologists were approached in their workplaces. Of these, 101 (48%) responded to the survey questionnaire, with a mean age of 39.1 years (±10.7 SD) and equal representation from both genders. All the respondents were using smartphone technology, wherein 94.1% used it to access medical information through various Internet search engines. The most prevalent applications used were medical and drug reference applications (69.3 and 66.3%, respectively). In all, 65% of the dermatologists were using smartphones to take clinical photographs of patients for special purposes, and this type of usage was significantly higher (*p* < 0.05) among females and those older than 40.

**Conclusion:**

This study concludes that smartphone technology has an impactful role in dermatology practice, and many of its functions can be employed to achieve better practice and better patient care. Recommendations are suggested for clinicians using smartphones in dermatology practice.

## Background

In this digital era, the use of smart technology, which includes smartphones, has become popular and widely used among medical professionals. Smartphone technology offers numerous features that medical doctors need in their clinical activities, including phone calls, built-in digital cameras, Internet access, telecommunication media and software applications designed for special purposes. Hence, medical doctors are attracted by the functionalities that smartphones offer due to their busy and routine work, which requires them to be available and active all the time.

The potential for smartphones to revolutionize medical practice has become a reality, particularly in specialities where visual examination is a principal step in assessing a medical case, including dermatology, plastic surgery, maxillofacial surgery and orthopaedics [1]. Dermatology is a speciality that inherently relies on visual examination for diagnosis, monitoring and treatment [2].

The smartphone as a device has similar capabilities to a personal computer or laptop and could play an important role in supporting medical practitioners in their clinical practice. Smartphones provide the user with Internet access to search the World Wide Web (WWW) for information from unlimited resources, which enables medical practitioners to utilise research regarding prognosis, diagnosis, treatment, etc. from different databases, such as PubMed, Scopus and Ovid, as well as other evidence-based resources.

Previous studies have found that the capabilities of smartphone technology in capturing, storing and sending data on skin lesions can help dermatologists to: (1) seek second opinions from consultants or peers (teleconsultation); (2) monitor diseases and treatments; (3) teach and educate residents and faculty students; and (4) conduct and publish research [1–3]. However, some crucial concerns have come to the fore regarding the use of smartphones concerning patients’ privacy and confidentiality [4, 5].

Some research studies have identified the use of smartphone digital cameras in facilitating remote consultation in dermatology, as well as in burn injuries [6–9]. The growing popularity of teleconsultation in healthcare is attributable to the technological advances in smartphones regarding digital imaging and data transmission [10].

It therefore becomes imperative to keep abreast of smartphone technology developments worldwide, and adopting the latest technology is equally important in medical practice to benefit from the technology’s advantages. Therefore, in medical practice, it is important to study users’ attitudes towards smartphone technology and verify its uses for patient care and safety. In Kuwait, generally, there is a lack of research into the use of smartphone technology in medical practice, particularly in dermatology. Hence, this research primarily is intended to study the role of smartphone technology use in dermatology practice in the governmental sector, as well as to assess the potential of digital photography and users’ concerns. The objectives of this research study were to: (1) identify the pattern of use of smartphone by dermatologists in their clinical practice; (2) explore the role of smartphone use in dermatology practice; and (3) identify the need for guidelines for the safe use of smartphones in healthcare organisations, especially for clinical photography, in regard to patients’ privacy and confidentiality.

## Methods

### Study design, research setting and participants

This research study was a cross-sectional survey, conducted from 4th February to 12th April 2018. It included all dermatology practitioners currently working in the governmental sector in Kuwait, namely: board residents, assistant registrars, registrars, senior registrars, specialists, senior specialists and consultants. The private sector was excluded from this study because it has its own work policy, unlike the governmental sector, which might have affected the results of the study. A survey questionnaire was used to collect information from practitioners in 11 specialized dermatology centers distributed across six health regions: Al-Asemah, Al-Sabah, Al-Farwaneya, Al-Jahra, Mubarak Al-Kabeer and Hawally. Dermatology care is provided through general hospital care (*n* = 4) at 15 clinics or through specialized centers (*n* = 7) at 25 clinics. A patient requires a referral from primary care or secondary care to obtain dermatological care. In Kuwait, healthcare services are provided to citizens for free and to expatriates for small charges.

### Research instrument

The questionnaire used in this survey study was validated by previous studies [3, 5] and consisted of four sections of items to assess: (1) the demographic characteristics of the participants (8 questions); (2) their attitudes towards their current use of smartphones (7 questions); (3) the difficulties and challenges associated with smartphone use (2 questions); and (4) concerns associated with clinical photography (8 questions), including the privacy and confidentiality of patients. The questions were closed-ended and provided in two response forms: single choice or multiple choice. Space was also provided for additional responses, if any. Ethical approval was elicited from the Ethical Research Committee at the Ministry of Health, Kuwait.

### Procedure

Pre-testing was conducted by distributing the questionnaire to ten practitioners to solicit their feedback on the suitability of the questions; accordingly, minor modifications were made to suit the research setting. The completed questionnaires were reviewed for accuracy and validity and tested for reliability, and Cronbach’s alpha was recorded as 0.767. All of the dermatology practitioners were approached during their working shifts and were verbally invited to take part in the study. The questionnaires were distributed personally to all available practitioners at the dermatology centers. Completed questionnaires were collected from the participants over five working days for each centre, giving busy practitioners the opportunity to respond and complete the questionnaire. The questionnaire was printed only in English and took 3–5 min to complete.

### Statistical analysis

Data management, analysis and graphical presentation were carried out using the computer software Statistical Package for the Social Sciences (SPSS), version 24.0 (IBM Corp.). The descriptive statistics are presented as numbers and percentages for the categorical variables. The quantitative variables (age and score of importance of smartphone technology in dermatology practice) are presented as means and standard deviations (SDs) with a 95% confidence interval (CI). The chi-square test and Fisher’s exact test were applied to find any associations or significant differences between the categorical variables. Student’s t-test and analysis of variance (ANOVA) were used to compare the mean ages and scores of different groups. A two-tailed probability value *p* < 0.05 was considered statistically significant.

## Results

Of the 323 dermatology practitioners working in the governmental sector in Kuwait, 210 were approached in their workplaces, and 101 completed the survey questionnaire, giving a response rate of 48%.

### Sample demographics

The mean age of the respondents was 39.1 (±10.7 SD), ranging from 20 to 72 years. Almost a third (30.7%) of the respondents were in the age group 30–39 years. There was almost equal representation of both genders: 50 males and 51 females (Table 1), though the mean age of the males was significantly higher (*p* < 0.004) than that of the females (42.2 ± 11.8 vs. 36.1 ± 8.7). Nationality wise, 60.4% were Kuwaiti nationals, and the remaining 39.6% were non-Kuwaiti Arabs, where the Kuwaitis were significantly younger (*p* < 0.001) than the non-Kuwaiti Arabs, (35.6 ± 9.0 vs. 44.5 ± 11.0 years). The study included all levels of dermatology practitioners: board residents (12.9%), registrars (58.4%) (including assistant and senior registrars), specialists (18.8%) and consultants (9.9%).

**Table 1:** Sample demographics (*n* = 101)

Demographics	No.	Percentage
Gender		
Male	50	49.5
Female	51	50.5
Nationality		
Kuwaiti	61	60.4
Non-Kuwaiti Arab	40	39.6
Age group (years)		
20–29	25	24.8
30–39	31	30.7
40–49	27	26.7
> =50	18	17.8
Mean (±SD) age/range	39.1 + 10.7	(20.0–72.0)
Practitioner level		
Board resident	13	12.9
Assistant registrar	14	13.9
Registrar	29	28.7
Senior registrar	16	15.8
Specialist	10	9.9
Senior specialist	9	8.9
Consultant	10	9.9

### Smartphone technology

All of the respondents used smartphone technology. The most commonly used operating system was iOS (49.5%), followed by Android (37.6%) and others (12.9%) (e.g. BlackBerry, Windows, etc.). The English language was used as the main medium by 91.1% of the users, followed by Arabic (5.9%) and others (2.9%). The majority (68.3%) of the respondents were self-taught users of smartphone technology: 8.9% had learnt from YouTube, blogs, etc. and 12.9% had learnt from their peers, while only 9.9% had learnt through proper training, like workshops and seminars. Of the respondents, 92.1% considered themselves computer literate: 51.5% said they had good skills, while 40.6% mentioned having basic skills only.

### Accessing medical information

In total, 94.1% of the dermatologists said that they used smartphones to access medical information through various Internet search engines, such as Google (75.2%), Medline (50.5%) and UpToDate (42.6%). The frequency of use for medical information was reported as daily (68.3%), weekly (22.7%), and rarely or never (9%). No significant difference (*p* > 0.05) was found in medical information access with respect to demographics.

### Medical applications

**Fig. 1:**
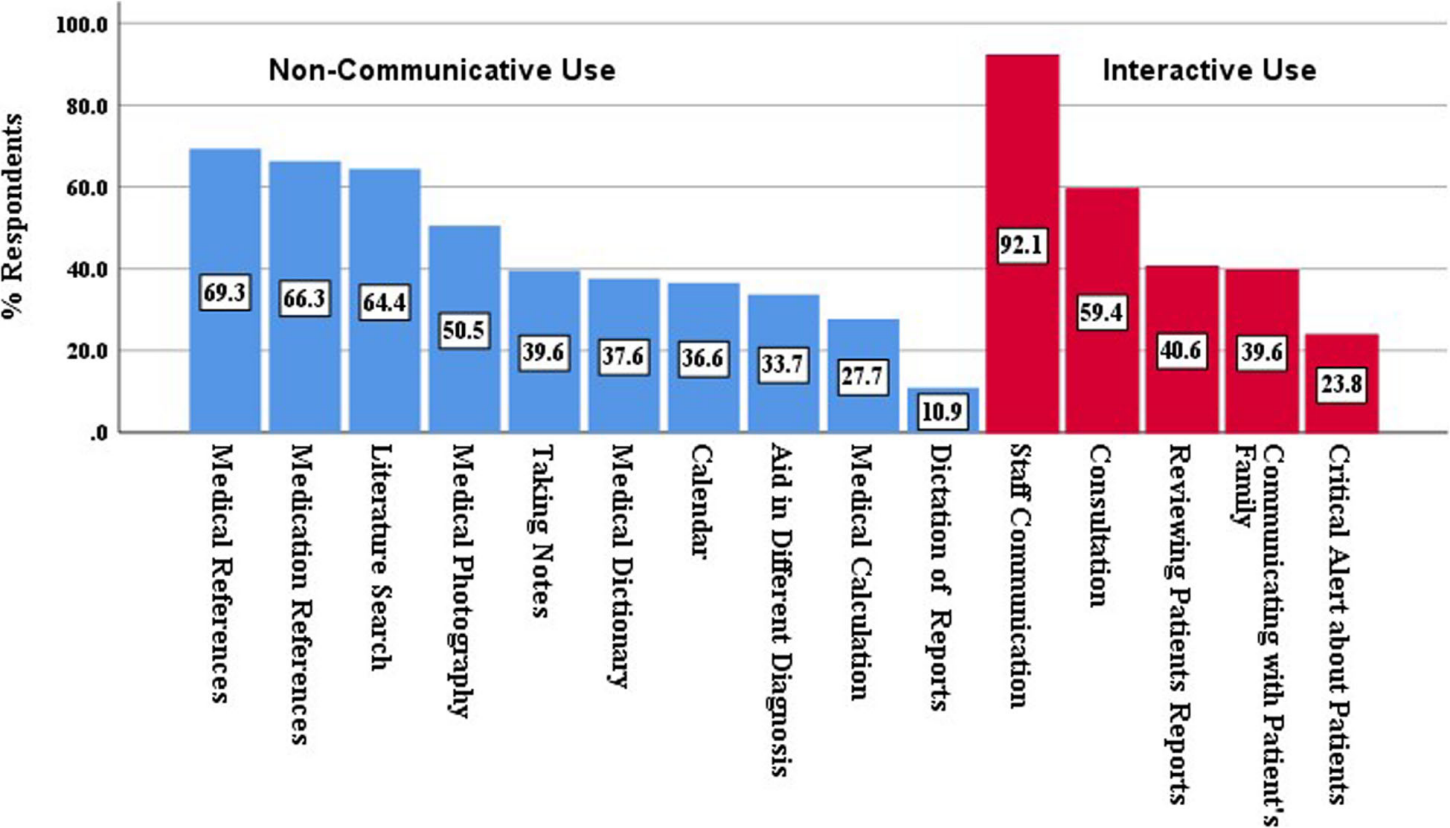
Use of non-communicative and communicative (interactive) applications

The most prevalent non-communicative medical applications used were medical references (69.3%), medication references (66.3%), literature searches (64.4%) and medical photography (50.5%) (Fig. 1). Interactive uses in medical practice were for staff communication (92.1%), consulting (59.4%), reviewing patient results (40.6%), communicating with patients’ families (39.6%) and communicating critical alerts about patients (23.8%). However, many general applications were also used, such as phone calls (88.1%), chatting (WhatsApp, etc.) (89.1%), personal email (Gmail, Yahoo, etc.) (77.2%), social networking (Facebook, Twitter, etc.) (64.4%), VoIP/video calls (Skype, FaceTime, etc.) (57.4%) and official work email (university, organisation, etc.) (29.7%).

### Safety of using smartphones in medical practice

The results show that 43.6% of the dermatologists were never concerned about mobile access and privacy, while 15% considered it daily. Regarding discussing patients’ data via smartphones, most of the practitioners reported that using personal email (61.4%), work email (51.5%) and short messages (52.5%) was safer and more secure than chatting applications and social networks, such as Facebook and Twitter.

### Clinical photography

**Fig. 2:**
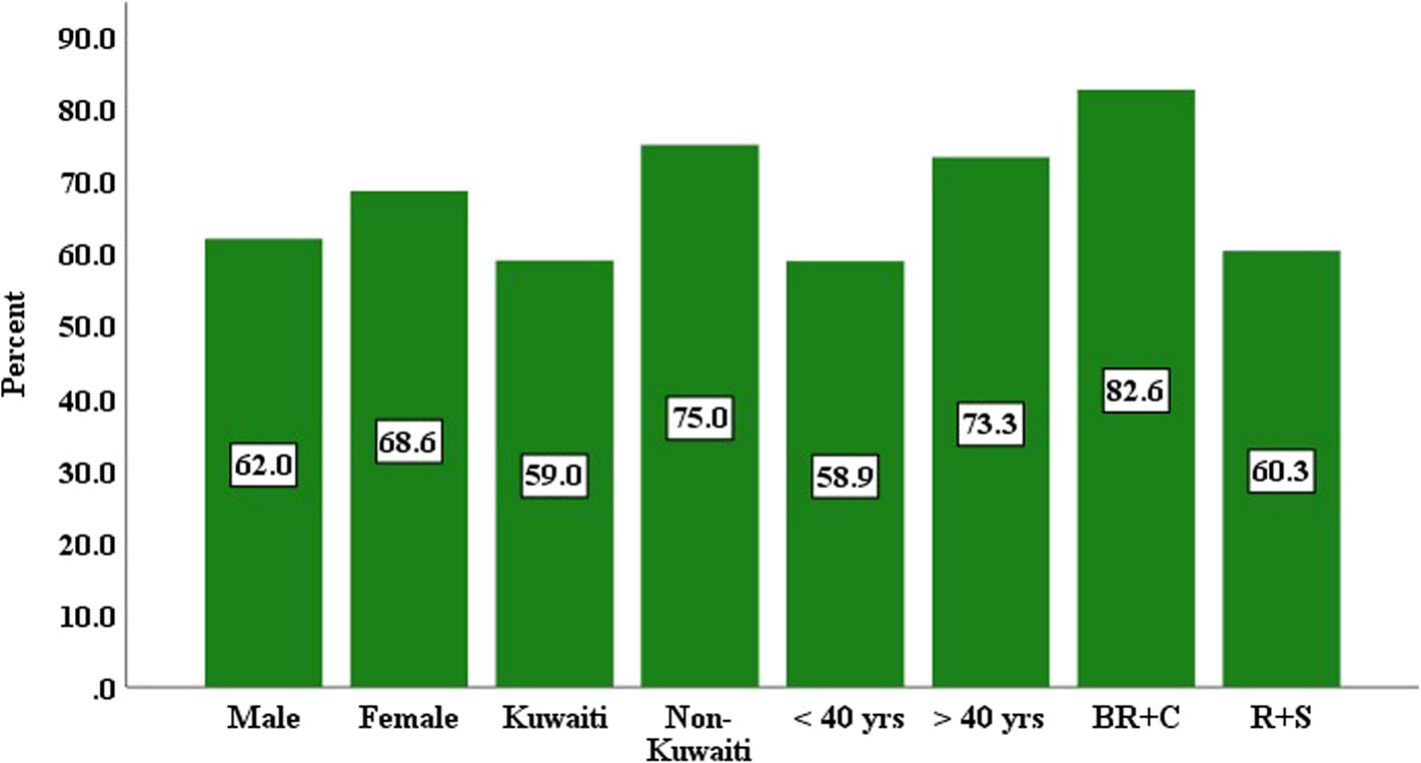
Clinical photography use (%) and respondents’ demographics BR = board resident, C = consultant, R = registrar and S = specialist

In all, 65% of the dermatologists mentioned using smartphones to take clinical photographs of patients for special purposes, such as for treatment or disease monitoring (56.4%), to gain advice from peers/consultants (55.4%), to share with colleagues (52.5%), for teaching purposes (40.6%) and for research or publications (21.8%). With respect to gender, nationality and age, smartphone clinical photography was more widely used by female, non-Kuwaiti dermatologists above 40 years old, and its use was significantly higher among board residents and consultants (Fig. 2). Females in particular were significantly more likely (*p* = 0.037) to use it to seek advice from consultants and peers (Table 2) compared to males, while those above 40 years old were significantly more likely (*p* = 0.024) than those under 40 years old to use it for disease monitoring. Board residents and consultants were significantly more likely to use it for teaching purposes than registrars and specialists were (60.9% vs. 34.6%, *p* < 0.024). In addition, texting/emailing pictures to colleagues for advice or second opinions was reported as occurring ‘often’ (32.7%), ‘occasionally’ (31.7%), ‘rarely’ (21.8%) or ‘never’ (13.8%).

**Table 2:** Clinical photography use and purpose

Clinical Photography: Use and Purpose	Gender M/F (50/51)	Nationality K/NK (61/40)	Age (yrs) ≤40/> 40 (56/45)	Position BR + C/R + S (23/78)
To take clinical photographs of patients	31/35	36/30	33/33	19/47
p-value	0.484	0.099	0.131	0.048
To gain advice from consultants	22/33	31/24	31/24	12/43
p-value	0.037	0.365	0.839	0.803
For treatment/disease monitoring	26/31	33/24	26/31	15/42
p-value	0.373	0.559	0.024	0.334
To share with colleagues	24/29	32/21	28/25	14/39
p-value	0.373	0.997	0.578	0.359
For teaching purposes	19/22	26/15	22/19	14/27
p-value	0.599	0.608	0.765	0.024
For research and publications	11/11	12/10	10/12	8/14
p-value	0.958	0.526	0.286	0.086

*M* = male, *F* = female, *K*=Kuwaiti, *NK* = non-Kuwaiti, *BR* = board resident, *C* = consultant, *R* = registrar and *S* = specialist

### Perceived importance of smartphone technology

**Fig. 3:**
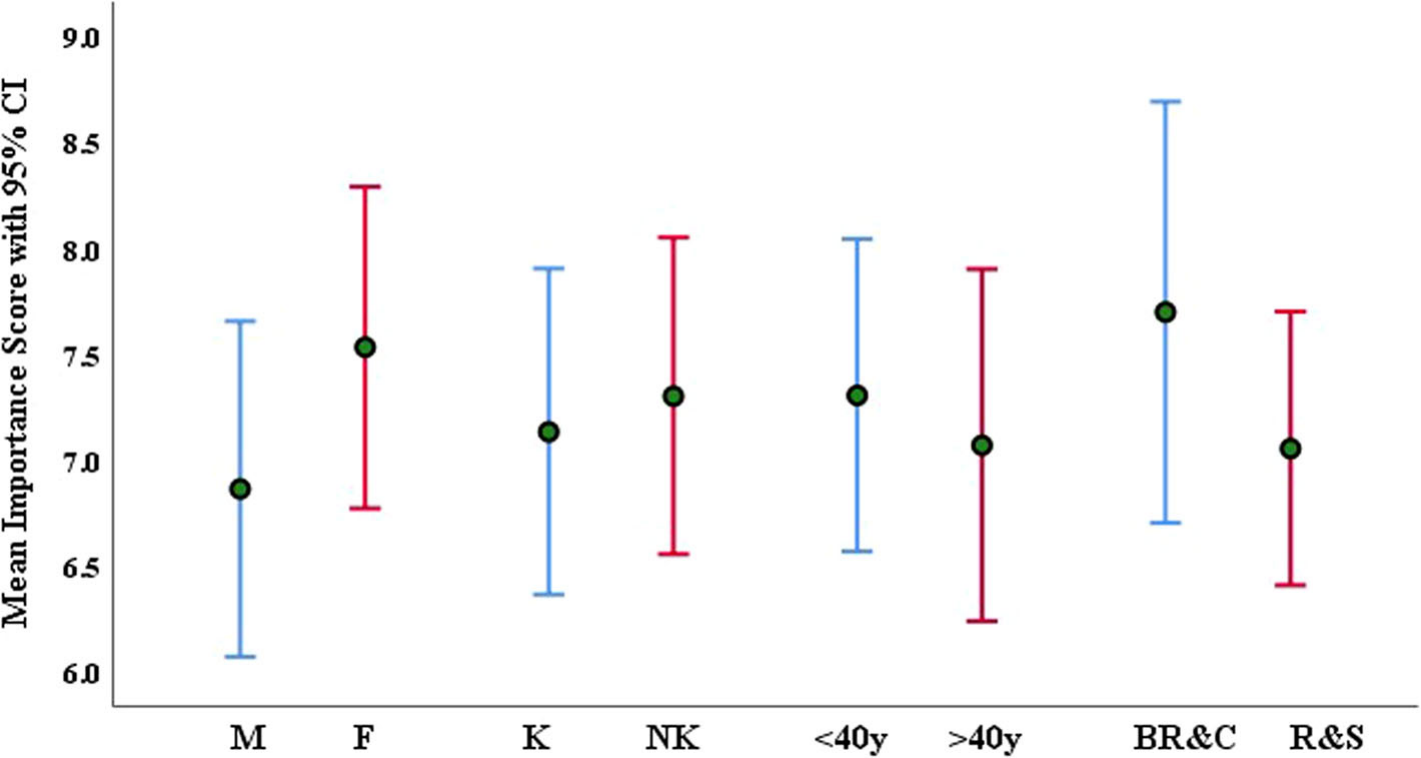
Perceived importance of smartphone technology (mean score with 95% CI) among the dermatologists, with respect to demographics. K=Kuwaiti, NK = non-Kuwaiti, y = year, BR = board resident, C = consultant, R = registrar and S = specialist

The respondents were asked to score (on a scale of 1–10) the importance of smartphone technology in dermatology practice with respect to different characteristics, and their mean (±SD) scores are presented in Table 3. The study found an overall mean score of 7.2 (±2.8SD) and a median score of 8.0. Females and younger dermatologists scored higher compared to males and older respondents, though no statistically significant difference was found. The scores of those who reported ‘Yes’ to clinical photography (using it for gaining advice, sharing with colleagues, teaching, treatment monitoring, research and publications) were significantly higher (*p* < 0.001) than those who gave a ‘No’ response. The mean scores with respect to demographics are presented in Fig. 3.

**Table 3:** Rated level of importance (Mean ± SD) of smartphone technology

Smartphone technology: use and purpose	Response	N (%)	Mean ± SD	*p*-value
To take clinical photographs of patients	Yes	66 (65.3)	8.41 ± 1.88	< 0.001
	No	35 (34.7)	4.91 ± 2.72	
To gain advice from consultants	Yes	55 (54.5)	8.60 ± 1.70	< 0.001
	No	46 (45.5)	5.52 ± 2.86	
For treatment/disease monitoring	Yes	57 (56.4)	8.23 ± 2.00	< 0.001
	No	44 (43.6)	5.86 ± 3.04	
To share with colleagues	Yes	53 (52.5)	8.40 ± 1.83	< 0.001
	No	48 (47.5)	5.88 ± 3.01	
For teaching purposes	Yes	41(40.6)	8.51 ± 1.78	< 0.001
	No	60 (59.4)	6.70 ± 2.95	
For research and publications	Yes	22 (21.8)	8.41 ± 1.18	< 0.001
	No	79 (78.2)	6.86 ± 2.97

### Difficulties and challenges

**Fig. 4:**
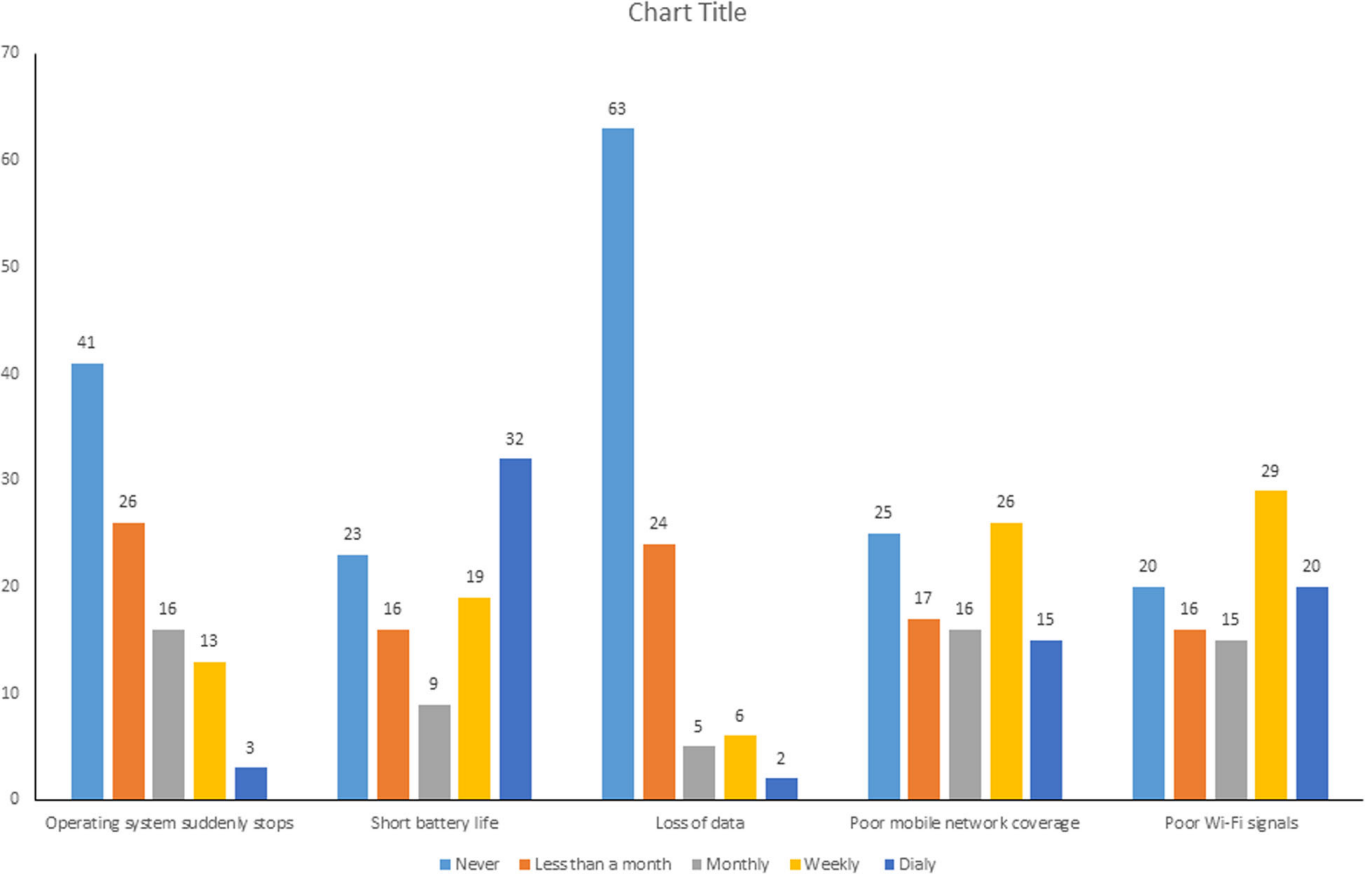
Distribution of smartphones’ difficulties faced by practitioners (in percentages)

The respondents were asked to rate the difficulties and challenges they face when they use the smartphones, using a Likert scale. The results showed that 32% of the dermatology practitioners considered short battery life a major difficulty faced daily. While 29% considered poor Wi-Fi signals as a difficulty faced weekly (Fig. 4).

**Fig. 5:**
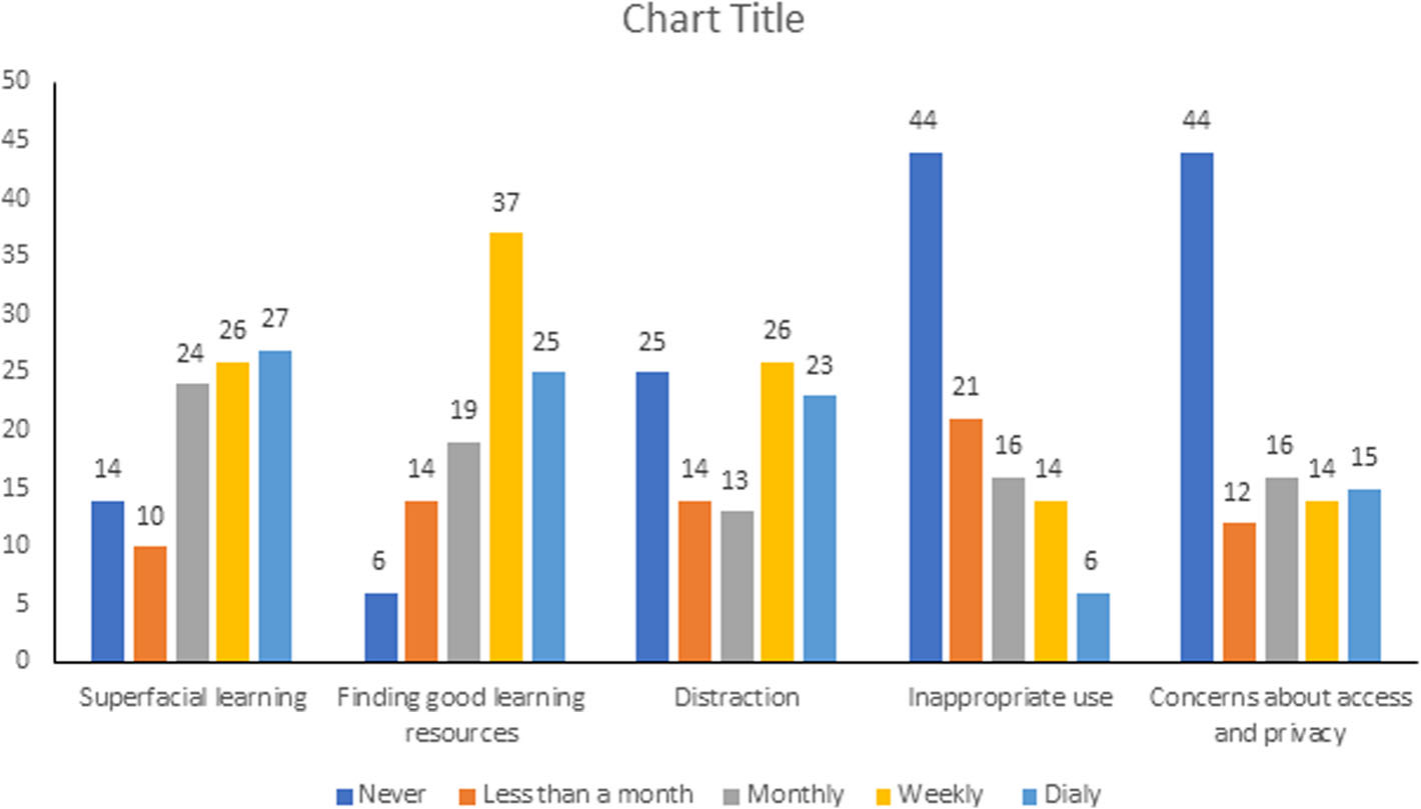
Distribution of practitioners’ challenges when using smartphones (in percentages)

Results showed that dermatology practitioners rated superficial learning (26%); difficulty to reach knowledge (27%), finding good learning resources (25%), and distraction (23%) as the greatest challenges to smartphone usage within the work environment (Fig. 5).

### Patients’ consent and privacy

Awareness of the requirement for gaining valid consent from patients was found among 77.5% of the respondents; 58.6% of the respondents reported the need for written consent, while 41.4% felt that only oral consent was needed. In all, 92% of the respondents reported discussing the proposed use of clinical photos with their patients, while only 76% mentioned ensuring patients’ awareness of their privacy rights. In addition, 86.9% of the respondents agreed with the need to take appropriate steps to ensure the security of stored photos.

## Discussion

It was found that smartphone use in dermatology practice was common, wherein the practitioners utilized several features of their smartphones, including using the built-in cameras for the clinical photography of skin lesions. Thus, it is crucial to consider smartphone usage in the official policy development of government hospitals in Kuwait.

### The use of smartphone technology in dermatology practice

The findings of this study show that all of the dermatologists owned smartphones, indicating a full adoption rate of 100%. This top level of smartphone ownership could be related to the fact that the majority of the respondents were computer literate (92.1%) and possibly liked to use technology, as most of them had learnt about smartphone use by themselves. These findings are consistent with those of previous studies in Saudi Arabia [3] and the United Kingdom (UK) [11]. In dermatology practice, some studies from Australia found that the use of smartphones for clinical practice was common [1, 5], exceeding 97%, particularly among junior practitioners, in which the sharing of clinical images using smartphones was practiced on a weekly basis (> 50%) [1]. In these studies, the focus was on junior populations, while this study covered all levels of dermatology speciality. However, it is obvious that smartphone ownership is widespread among medical professionals, regardless of age. Smartphones are considered an excellent tool to maintain communication in different forms while on the go, perhaps to save lives [12].

Furthermore, the findings reveal that the dermatology practitioners utilized smartphones in their clinical practice, wherein 94% accessed the Internet to search for information from different medical databases, such as Google and Medline; 68.3% of them did this on a daily basis, with no differences in regard to demographics (age, gender and nationality). These findings were consistent with the findings of a UK study conducted among surgeons [13], in which 86.2% of the respondents (275 of 319) used smartphones to access search engines, mostly Google, with 42.2% of them doing this on a daily basis (116 of 275). Accessing the Internet to seek medical information is very crucial in dermatology practice because it influences the type and speed of decision-making for doctors’ practice and patients’ care, as previous studies have reported [11, 14].

The most prevalent non-communicative medical applications used by the dermatology practitioners were, unsurprisingly, medical references (69%) and drug references (66%) to retrieve medical and drug information quickly, particularly at the point of care. These applications are useful not only for dermatology practitioners but for other medical specialities as well, as reported in a Saudi Arabian study of medical residents, with 70–100% of respondents utilizing drug reference applications [3]. In a Turkish study, more than 50% of family medical practitioners used drug reference applications [15]. In the UK, the British National Formulary was the most common drug reference application used among surgeons (40.5%, 70 of 173), followed by eLogbook and MedCalc [13].

There are several applications designed to help dermatologists to diagnose clinical cases and make decisions, some of which are available as free downloads. The findings of this study show that only a third of the respondents (33.7%) were using these applications, so most of the respondents were not aware of or interested in the applications offered, or they did not see their usefulness in practice. In a UK study, medical doctors responding to an open-ended question indicated that they preferred to stick with the hospital guidelines for using highly recommended smartphone applications [11]. A systematic review conducted in 2018 into melanoma detection concluded that smartphone applications with store-and-forward techniques were useful for obtaining consultations over distance; however, verifying the quality of e-applications to ensure safety and efficiency is very important [16]. Therefore, it is apparent that smartphone technology can make a difference in healthcare delivery, but it is crucial to assess smartphone capabilities and the quality of applications, taking safety into account.

It was found that the majority of the respondents utilized interactive applications in their medical practice, including phone calls, messages (e.g. WhatsApp) and personal email (e.g. Yahoo and Gmail). Comparable results were reported in previous studies among medical doctors in Saudi Arabia [3, 17] and Turkey [15].

In this study, it’s found that short battery life of the smartphone was the greatest difficulty faced by dermatology practitioners. Similar difficulty was reported by medical doctors in Saudi Arabia [3]. Also, the findings of this study revealed that superficial learning and finding good learning resources with smartphones were more challenging to dermatology practitioners than distraction, which was rated as third most challenge. Similar results were confirmed in a qualitative study in Canada among medical teachers and learners [18]. In Saudi Arabia, distraction was reported as a major challenge among medical residents [3] as well as health care providers [19]. In a UK study, numerous challenges associated with smartphones use in surgical practice were acknowledged by surgeons, including misuse of smartphones within work environment, distraction, and contamination concerns [13]. Accordingly, safety of the patient should be taken into account by smartphone users in clinical practice.

### Clinical photography using smartphone technology in dermatology practice

It is known that the practice of dermatology relies on the visual examination of skin lesions for clinical diagnosis. This study found that more than half of the dermatology practitioners used smartphones to photograph the infected areas of patients for different purposes, mainly for monitoring treatment or disease progress and to obtain advice from peers or consultants. These findings are consistent with the findings of an Australian study into dermatology practice [1], as well as in other practices in medicine [3, 20]. The benefits of smartphones in the clinical practice of dermatology are apparent. The use of smartphones’ built-in cameras for diagnosing skin problems is increasing among practitioners, which could pertain to the ease with which users can not only take photos but can also store, send and receive captured images. However, clinical photography in dermatology requires paying special attention to camera capabilities regarding zooming and the resolution needed for taking clear photos to show skin texture, pigmentation and features [2, 21].

In this study, it was found that females, non-Kuwaitis, those above 40 years old, dermatology residents and consultants were the most likely to use smartphone cameras clinically. It seems that the job level of the respondents influenced whether they took photographs of patients’ skin lesions: dermatology residents study the photographs, while consultants use them to monitor the progress of treatment/disease and for teaching purposes. While this study found that female dermatologists were more likely to use smartphone cameras than males, previous studies found that gender had no influence on smartphone use [3, 13]. This study’s findings could be due to the influence of cultural factors in Kuwait that female doctors have fewer restrictions in photographing the skin lesions on patients’ bodies of both genders than male doctors do, except the sensitive areas of men, so they use the cameras more. A previous study reported that photographing is considered acceptable when written consent is provided and when the photograph is taken by a doctor of the same gender with a hospital-owned camera [22].

Interestingly, the findings of this study reveal that 80% of the respondents (mostly female and young) rated using smartphone technology in dermatology practice as important. Several previous studies have confirmed that the current generation of medical students, junior doctors and others are expected to have high familiarity with mobile technology and high computer competency and tend to adopt digital practices to provide the best healthcare easily and swiftly [1, 3, 11, 23]. Furthermore, the multi-tasking ability of smartphone technology on the go in one mobile device is the main reason for healthcare practitioners using it.

### Privacy and confidentiality of patients’ information

In this study, it was found that smartphones’ accessibility and the privacy of data were not considered concerns by most of the practitioners. At the same time, they reported that using email to discuss patients’ data was safer than using social networks. Similar results have been found in previous studies [3, 24], in addition to other concerns, such as breaches of privacy and confidentiality and mistaken data inputs. It seems that the respondents were unaware that security applications can be installed for the safer use of smartphones, providing similar security to email. Hence, new guidelines on using smartphones safely need to be developed. Additionally, the security of smartphone devices needs to be considered to ensure patients’ privacy and confidentiality.

The findings of this study show that approximately three-quarters (77.5%) of the respondents were aware of the need for patient consent prior to taking or storing images with smartphones. Some of the dermatology practitioners believed that written consent was required, while others only asked for oral consent. It seems that the infected area of the body (the disease), the procedure (e.g. taking a biopsy of a skin lesion), and the gender and age of the patient influenced the perceived need for written or oral consent or both. As some of the practitioners considered photographing a skin lesion to be a routine dermatology practice, the oral consent of a patient was deemed sufficient. A previous study into dermatology practice and the law concluded that photographing using smartphone cameras is increasing and that seeking consent from patients in a professional and liable way is important to maintain their rights (1, 5).

The findings show that the majority of the respondents (76%) stated that patients were aware of their privacy rights, although some patients were unaware of their privacy rights or did not trust their doctors. In a US study that surveyed 300 patients about using smartphone cameras for medical photography, patients preferred the use of hospital-owned devices, rather than the doctors’ personal devices. Moreover, the study concluded that patients’ preferences should be taken into consideration [25]. In the present study, the researchers did not find hospital guidelines on smartphone use regarding maintaining the legal rights of both the doctor and the patient, while other studies have reported the existence of such guidelines in the workplace, including for clinical photography [1].

### Limitations

This study was limited to dermatology practitioners working in the governmental sector and their use of smartphone technology, excluding other smart technology, such as tablets and personal computers. In addition, the response rate of the dermatology practitioners was low (48%). Future studies are recommended to study the perceptions of patients and policymakers alike to explore their concerns, including in the private sector.

## Conclusion

This study found smartphones were widely used in dermatology practice and that the degree and manner of use varied by practitioner demographics, wherein many of smartphone functions can be employed to achieve better practice and better patient care. However, to ensure good and safe use of smartphones, guidelines should be developed to standardise the clinical practice in healthcare facilities and to maintain the legal rights of dermatologists and patients alike.

### Recommendations

The results of this research study confirm that the use of smartphone technology becomes popular among dermatology practitioners; the extant literature shows similar results among other medical specialities. Hence, it is of paramount importance to develop guidelines at the organisational level for the safe and effective use of smartphones in dermatology practice, for which the following recommendations are suggested:

- Maintaining patient privacy and confidentiality is not only a necessity in medical practice but also part of codes of ethics, which every individual in a healthcare facility must abide by. The legal use of personal smartphones in clinical practice, therefore, requires patients’ consent, particularly when taking photographs of their bodies. Consent for taking photographs should be sought to maintain the legal protection of both the practitioner and the patient. To ensure this, a signed consent form should be stored with the photographs in the patient’s record. In the case of electronic records, a secure smartphone application can be used to incorporate smartphone photographs into the records to transmit all patient information, keeping patients’ records complete.
- For the better use of smartphones in dermatology, photographs must be taken of infected areas only (with no patient identity information) and written consent should be sought from the patients, including under-aged patients, who require parental consent. Additionally, the identity of the patient must be kept anonymous, attempting not to show scars or tattoos.
- For the safer use of smartphone technology, (1) high-security applications should be recommended for smartphone clinical use, downloaded through an online store that provides full protection of users’ information; (2) practitioners should store clinical photographs in secure areas on their smartphones and not for a long time (i.e. until they have been transmitted to the patient’s record); (3) breaches of patients’ privacy and confidentiality by practitioners would be vulnerable to legal accountability, and practitioners should be aware of this; and (4) personal conversations between senders and receivers through chatting applications should be clarified for confirmation and to avoid mistaken data inputs.
- In clinical practice, safety of the patients should be considered in regards to the smartphones use: (1) practitioners should be aware about trusted resources to obtain medical information via smartphones; (2) practitioners should avoid the distraction caused by the smartphones, particularly at the point of care to maintain the confidence between doctor and patient; also (3) attention should be given to avoid contamination when using smartphone [13], and use of barrier film to cover the device and control the infection.
- The medical board of each medical speciality should make recommendations on the best smartphone applications for clinical use. In addition, issues with technology and smartphone use should be considered and addressed regularly in meetings to find the best usage protocol to organise the work inside the healthcare facility, such as in the case of teleconsultation. Involving health informatics specialists, who specialise in both healthcare and technology, would contribute to the professionalisation of this situation.
- The Kuwait Medical Association should play a role in supporting the digitisation of medical practice, should help the medical community to adopt smart technology successfully and should prepare Kuwaiti society to adapt to this advanced technology.
- Information technology support should be offered 24/7 on smartphone use in clinical practice, including offering a Wi-Fi connection, providing workshops on using smartphones properly and resolving any technical issues.
- Patients should be made aware of the benefits of smartphone use by healthcare practitioners in treatment, education and research, and patients should be informed of their legal rights regarding privacy and confidentiality.
- Senior management should encourage medical practitioners to use the highly recommended smartphone applications for clinical use through paying the fees for these applications. Importantly, the quick guide for store-and-forward teledermatology and live/interactive teledermatology developed by the American Telemedicine Association [26] should be adopted.
- The experience of using smartphone applications in clinical practice should be explored by conducting research, offering the opportunity to identify the advantages and disadvantages.

## Data Availability

The datasets used and/or analysed during the current study are available from the corresponding author on reasonable request.

## Abbreviations

CI: Confidence interval
SD: Standard deviation
SPSS: Statistical Package for the Social Sciences
WWW: World Wide Web
